# Late diagnosis of Fabry disease caused by a de novo mutation in a patient with end stage renal disease

**DOI:** 10.1186/s13104-015-1696-5

**Published:** 2015-11-24

**Authors:** Antonio Pisani, Aurora Daniele, Carmela Di Domenico, Ersilia Nigro, Francesco Salvatore, Eleonora Riccio

**Affiliations:** Chair of Nephrology, Department of Public Health, University Federico II of Naples, Naples, Italy; CEINGE-Advanced Biotechnology S.c.a r.l., Via Gaetano Salvatore 486, 80145 Naples, Italy; IRCCS- SDN Fondation, Via Emanuele Gianturco 113, 80142 Naples, Italy

**Keywords:** Fabry disease, Alpha-galactosidase A, GLA gene, De novo mutation

## Abstract

**Background:**

We present the case of a white 35-year-old male with a diagnosis of Fabry disease and negative family history.

**Case presentation:**

At the age of 31, he underwent a renal biopsy with a diagnosis of hypertension-induced nephroangiosclerosis. At the age of 35, he was referred to our hospital and started dialysis: the unusual finding of left ventricular hypertrophy with a normal ejection fraction and of myocardial fibrosis at the cardiac magnetic resonance suggested a diagnosis of Fabry disease, although there was no apparent family history—so extensive tests were subsequently undertaken. The patient had low plasma levels of α-galactosidase A and the genetic analysis showed a single nucleotide point mutation in hemizygosis at nucleotide c.901 C>T in exon 6 of the GLA gene, confirming the diagnosis of Fabry disease. We extended the genetic analysis to all family members of the patient (mother, sister and brothers) and none of them had any alteration in the GLA gene, suggesting a de novo mutation in the patient.

**Conclusions:**

In a family, it is rare to find only one Fabry disease affected subject with a de novo mutation. These findings emphasize the importance of early diagnosis, genetic counseling and studying the genealogical tree of suspicious patients, even in absence of a typical family history.

## Background

Fabry disease (FD) (OMIM: 301500) is a rare X-linked hereditary disease (incidence 1:117.000 live births) caused by deficiency of the lysosomal alpha-galactosidase A enzyme (αGalA; E.C. 3.2.1.22) [1]. Human αGal A is a homodimeric glycoprotein with a molecular weight of 46 kDa. GLA gene is located on Xq22.1, spans approximately 13 kb of genomic DNA and contains seven exons [2, 3]. As a consequence of the αGal A deficiency, neutral glycosphingolipids, mainly globotriaosylceramide (Gb3), accumulate in a variety of cells and tissues, leading to a wide clinical spectrum of clinical manifestations [4]. Evolving knowledge suggests that FD has a wide spectrum of heterogeneous phenotypes, which ranges from the classical phenotype with kidney, cardiovascular and cerebrovascular disease [5], to a seemingly asymptomatic disease, occasionally observed in females [6].

To date, over 600 mutations are described in the human gene mutation database (http://www.hgmd.org) as causative of FD. Moreover, most mutations are “private”, occurring only in single pedigrees, while mutations at CpG dinucleotides have been identified in unrelated families [7]. Correlation between genotype and residual enzyme activity is not strong, depending on the mutation and additional genetic and non-genetic factors. Furthermore, very few “de novo” mutations have been detected with a frequency still unclear [8].

Here we report a case of non-diagnosed FD patient until the age of 35 without a familial history of the disease; we performed both clinical and molecular diagnosis identifying a de novo mutation.

## Case report

The patient, a white 35 years old man, had been healthy until the age of 31 years, except for mild articular pain and hypohidrosis since childhood. His father died of pulmonary enphysema at the age of 54 years, and had no renal or cardiovascular disease. His mother, 60 years old, has hypertension and a past history of ischemic heart disease. The patient has two brothers aged 38 and 33 years and a sister aged 27 years, all perfectly healthy.

At the age of 31 years, he was hospitalized for proteinuria in nephrotic range (6 g/24 h), hypertension and a reduced creatinine clearance rate (eGFR 57 mL/min). He underwent renal biopsy, with a diagnosis of nephroangiosclerosis. The biopsy material contained 20 glomeruli: global glomerular sclerosis, due to ischemic damage, was demonstrated in fourteen glomeruli, and the remaining six glomeruli showed only minor abnormalities; arterial and arteriolar hyalinosis were also present. Signs of tubular atrophy, interstitial fibrosis, and interlobular sclerosis were noted. Immunofluorescence examination did not show deposition of IgA, IgG, IgM, C1q, C3, k, and lambda fragments. Electron microscopy was not performed. At that time, the echocardiography showed concentric left ventricular hypertrophy (LVH) (ejection fraction 64 %) and an abdominal ultrasound showed no abnormalities. The patient was discharged with diagnosis of hypertension-induced nephroangiosclerosis and started treatment with ACE-inhibitors.

At the age of 35, because of the fast progression of renal disease and the need to start dialysis, the patient was referred to our hospital. On admission, his blood pressure was 120/85 mm Hg, pulse was 72 beats/min and body temperature was 36 °C. Physical examination of the head, face, abdomen and extremities showed no abnormalities; moreover, there were no abnormal neurological findings.

Full blood count was normal. The values of blood biochemical parameters were urea 210 mg/dL, creatinine 9.4 mg/dL, total protein 8.0 g/dL and albumin 4.3 g/dL. Electrolytes and the result of liver function tests were normal. Twenty-four-hour protein excretion was 4.3 g, and his creatinine clearance was 9.3 mL/min. On admission, we also repeated abdominal ultrasound, which showed bilateral increased echogenicity, with a decrease in mean cortical thickness and loss of cortico-medullary definition. The echocardiography confirmed the concentric LVH with a normal ejection fraction (63 %). Cardiac magnetic resonance imaging (MRI) was subsequently performed to better evaluate the cardiac involvement, that showed a prolonged myocardial T2 relaxation time at the level of the inferolateral wall, secondary to myocardial fibrosis.

**Fig. 1:**
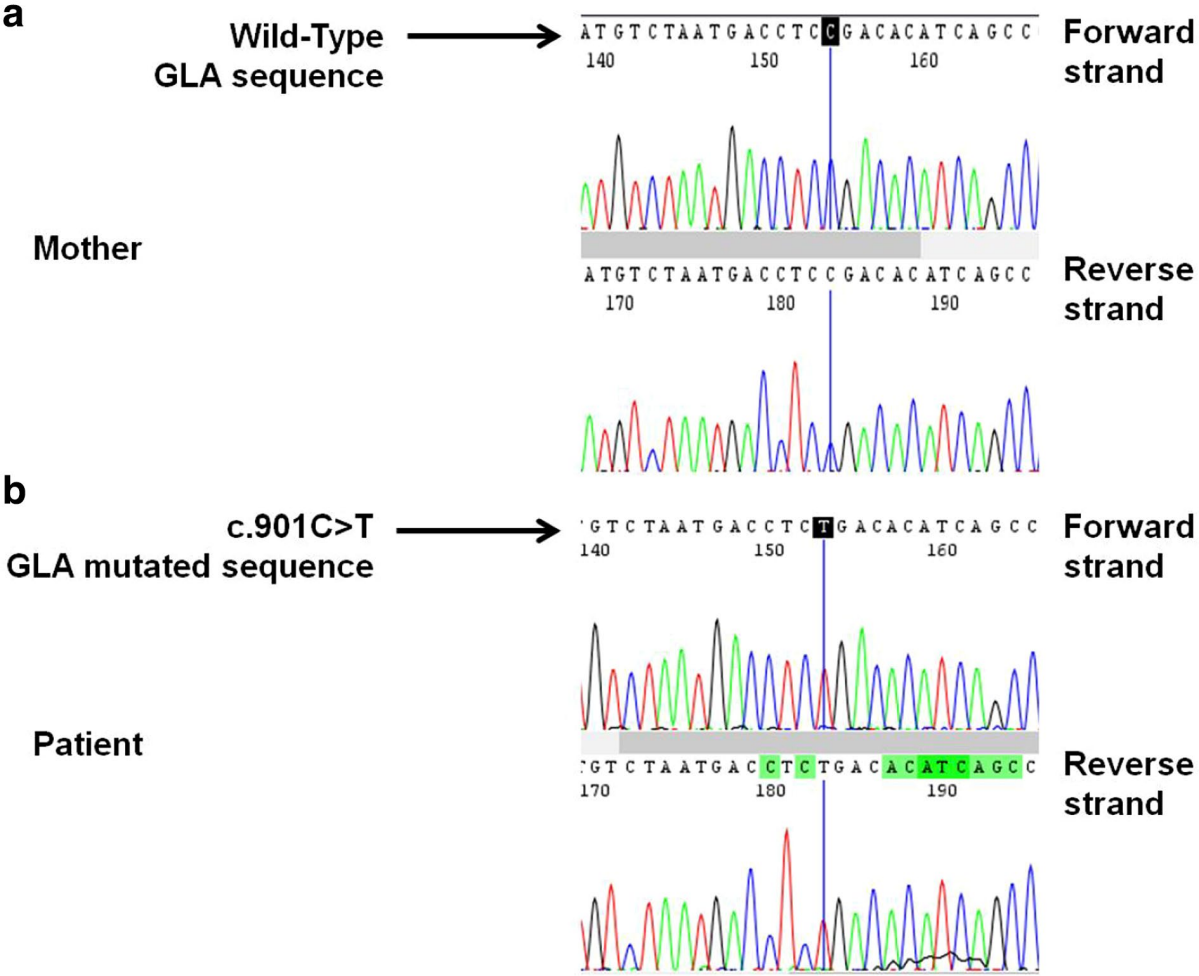
Molecular characterization of the c.901 C>T mutation in GLA gene. Sequence analysis of GLA exon 6 in our patient. The *top* electropherogram is from patient’s mother (**a**), the *lower* shows the patient’s mutation (**b**). The *vertical bar* indicates the position of the mutation

On the basis of these evidences and of a more accurate anamnesis, typical symptoms of FD—neuropathic pain, hypohidrosis, LVH, proteinuria and CKD—were identified, although there was no apparent family history of FD. Therefore, extensive tests were subsequently undertaken: evaluation of levels of α-Gal A in the plasma and molecular analysis were performed. Despite the finding of Malta crosses in the urine is a very common finding in Fabry disease, a urinalysis was not performed. The patient had low levels of α-Gal A (0.5 nmol 4 MU/mL/h; normal range 8–19 nmol 4 MU/mL/h); sequence analysis of the GLA gene (Fig. 1) showed a single nucleotide point mutation in hemizygosis at nucleotide c.901 C>T in exon 6 (p.Arg301X). We extended the genetic analysis to all family members of the patient (mother, sister and brothers) and none of them had any alteration in the GLA gene, suggesting a de novo mutation in the patient.

## Discussion

FD is an X-linked disease in which mutations of the *GLA* gene result in a deficiency of the enzyme α-galactosidase A and subsequent progressive, intralysosomal deposition of undegraded glycosphingolipid products, primarily Gb3, in multiple organs [1, 2]. The initial signs and symptoms of FD emerge during childhood and adolescence; however, because these signs and symptoms are not specific of FD, these patients are frequently misdiagnosed, and the correct diagnosis may be delayed. Major organ involvement typically occurs between the age of 20 and 30 years and result in a significantly decreased quality of life for both heterozygous females and hemizygous males, with lifepan typically reduced by approximately 15–20 years.

For the effective management of FD, an early diagnosis is required, and an early start of treatment remains essential in order to reduce the progression of the disease [2].

Due to its rarity and heterogeneity of the symptoms, FD clinical diagnosis remains a diagnostic challenge. In addition, the diagnosis is even more complex when family history is absent.

Here, we report a case of late diagnosis of FD in a patient of 35 years with progressive renal disease. The late diagnosis is mostly due to the absence of family history and of ultrastructural analysis of the renal biopsy. However, the clinical history and the finding of the characteristic cardiac abnormalities suggested the presence of FD, confirmed by the low α-Gal levels [3, 4] and the molecular analysis.

In fact, the molecular diagnosis, performed by sequencing the seven exons, the exon–intron boundaries and the promoter region of the GLA gene in the patient, revealed in the patient the c.901 C>T (p.R301X) mutation. This mutation, identified as a pathogenic mutation in 1995, inserts a stop codon in the aGalA protein and is reported to be responsible for the classical phenotype [5–9]. Moreover, it involves CpG dinucleotides, which are hot-spots for mutation [7].

The study of the relatives of the patient showed, surprisingly, that none of his family members (mother, sister and brother) was affected by the disease; all of them showed normal enzymatic activity and the wild-type GLA gene. This indicated that the disease was caused by a de novo mutation, arisen spontaneously in this male patient. To confirm these findings, the analysis of the genetic profile of the patient and his relatives was carried out, proving that they had a family relationship.

To date, more than 600 mutations have been identified in human GLA gene that are responsible for FD, including missense and nonsense mutations, small and large deletions (Human Gene Mutation Database, http://www.hgmd.org). Such mutations are usually inherited and cases of de novo onset, i.e. arisen spontaneously, occur rarely [10]. De novo mutation may be due to maternal germline mosaicism or spontaneous mutation [8, 10] and are very difficult to diagnose. In the literature, only few cases of de novo mutations in FD have been described [8–11], for the difficulty to recognize them. In one, the mutation c.493 G>C in the third exon of the GLA gene was detected in a 44-year old male patient with classical clinical manifestations of the disease and negative family history and in his 11-year old daughter [8]; another case described a patient with a de novo diagnosis of point mutation (R301X) in the GLA gene [9]. Other two studies described the cases of a 32-year old female and a 41-year old male, in which the de novo point mutation at position 691 of exon 5 and G373D, respectively, were detected [10, 11].

## Conclusions

Concluding, a strategy for timely FD diagnosis is recommended and genetic testing would be performed in suspicious patients even in absence of a typical family history to make an early diagnosis.

## Consent status

Written informed consent was obtained from the patient for publication of this case report and any accompanying images.

## Abbreviations

FD: Fabry disease
αGalA: α galactosidase A
Gb3: globotriaosylceramide
eGFR: estimated glomerular filtration rate
LVH: left ventricular hypertrophy
MRI: magnetic resonance imaging
